# The FDA authorization of direct-to-consumer genetic testing for three *BRCA1/2* pathogenic variants: a twitter analysis of the public’s response

**DOI:** 10.1093/jamiaopen/ooz037

**Published:** 2019-09-17

**Authors:** Megan C Roberts, Caitlin G Allen, Brittany L Andersen

**Affiliations:** 1 The National Cancer Institute, Division of Cancer Control and Population Sciences, Rockville, Maryland, USA; 2 Department of Behavioral Sciences and Health Education, Emory University Rollins School of Public Health, Atlanta, Georgia, USA; 3 Boston University College of Communication, Division of Emerging Media Studies, Boston, Massachusetts, USA

**Keywords:** breast cancer, genetic testing, social media, FDA, direct-to-consumer testing

## Abstract

**Objectives:**

In March 2018, the Food and Drug Administration (FDA) announced its authorization of a direct-to-consumer (DTC) genetic test for three pathogenic *BRCA1/2* variants. We sought to determine to whether social media discussion increased following the authorization, who was driving social media conversations, and what topics were discussed.

**Methods:**

Using Crimson Hexagon, we described tweets before, during, and after the FDA announcement authorizing 23andMe to return *BRCA1/2* results (3/4/18–3/10/18). We conducted qualitative coding of a subset of 605 tweets to better understand Twitter communication.

**Results:**

We identified 11 055 twitter posts across the week of FDA’s announcement. Twitter discourse about 23andMe and the FDA authorization peaked the day following the FDA’s press release. Most tweets (48.6%) were informational and 26.3% were either expressing opinions (about 23andMe and/or FDA authorization, 14.9%) or testimonials (personal experiences with genetic testing, 11.4%). The types of tweets varied over the week-long period (*P <  *.001).

**Discussion:**

Twitter discussion about the FDA’s authorization of DTC for three pathogenic *BRCA1/2* variants increased immediately following the announcement. As more genetic technologies are brought to the DTC market, social media sites, like Twitter, will play a role in disseminating this information, providing a platform for information exchange, consumer testimonials, opinion pieces, and research.

## BACKGROUND AND SIGNIFICANCE

Despite a previous warning letter to 23andMe, Inc.^1^ to stop the marketing of their product for the diagnosis of disease in 2013,^2^ the Food and Drug Administration (FDA) authorized the first direct-to-consumer (DTC) test by 23andMe, Inc. for pathogenic *BRCA1/2* variants in March 6, 2018. While there are over a thousand pathogenic variants associated with elevated risk of hereditary breast and ovarian cancer, this test provides results for three pathogenic variants that are most commonly found among those with Ashkenazi Jewish decent.^3^ This FDA authorization marks the first time consumers can use DTC testing for pathogenic variants related to cancer risk in the United States without a doctor’s order.

While public uptake of DTC genetic testing has increased over time,^4^ understanding of these tests remains low; in 2014, only 38% of people in the United States were aware of DTC testing.^5^ Recent studies show that people learn about potential harms and benefits of health applications, such as DTC testing, from social media and the internet.^6^ Events in the news, such as the FDA announcement or a celebrity health disclosure, may increase awareness about health applications. As an example, in May 2013, Angelina Jolie described her experiences with *BRCA1/2* testing and subsequent risk management in the *New York Times*, which prompted a 112% increase in internet searches about *BRCA1/2*.^7^ It is unknown to what extent the FDA announcement may have impacted online discussion about DTC testing, and what type of information was shared.

## OBJECTIVES

Thus, the objectives of this study were to learn more about (1) who engaged in social media discourse via Twitter surrounding the FDA announcement about 23andMe, (2) whether discussions of this testing increased over the period, and (3) what topics were discussed.

## MATERIALS AND METHODS

### Data source

We used the social media analysis tool Crimson Hexagon (Boston, MA, USA),^7^^,^^8^ to examine public engagement in Twitter discussions about 23andMe spanning before, during, and after the FDA’s announcement of its authorization for the company to offer select *BRCA1/2* test results (3/4/2018–3/10/2018). Our search criteria included related hashtags (#FDA AND (#BRCA OR #BRCA1 OR #BRCA2)). We also included keywords: “23&me” OR “23 & me” OR “23andme” OR “23 and me.” The selection of hashtags and keywords was based on relevancy to our topic of focus. Generalizable terms such as “cancer” or “variant” were excluded to better ensure tweets would be directly related to the FDA’s announcement.

### Measures and analyses

Outcomes of interest included the number of tweets overtime and the top influencers (i.e., users who drive conversations around topics and have high post engagement rates, such as retweets) who engaged in discourse about *BRCA1/2* and the FDA announcement. To obtain data on the content of tweets, we conducted qualitative coding for a random subset of tweets (*n* = 605) until we reached saturation of themes. Two authors double-coded 10% of the qualitative subset using a preidentified set of codes, compared coding, adjusted the codebook, and then independently coded remaining tweets after consistency of coding was reached. The final codebook included codes for whether the tweet was informational, a testimonial (i.e., personal story about using 23andMe), an opinion piece (i.e., opinions about 23andMe, excluding testimonials), research-related, other, or “colloquial” (i.e., 23andMe used in everyday lexicon). We also coded the sentiment of testimonials and opinion pieces as positive (i.e., only discussed the benefits of 23andme), negative (i.e., primarily discussed drawbacks of 23andme), or neutral (i.e., discussed both benefits and drawback or provided neutral information). We also sought to identify inaccurate information (among tweets coded as “informational”) about 23andMe and the FDA decision, but did not identify inaccurate information in our subset. We compared how the “type of tweet” varied before-, during-, and after- the FDA announcement using chi-square tests. Similarly, we examined sentiment of testimonials and opinion pieces by time of tweet.

## RESULTS

**Figure 1.**
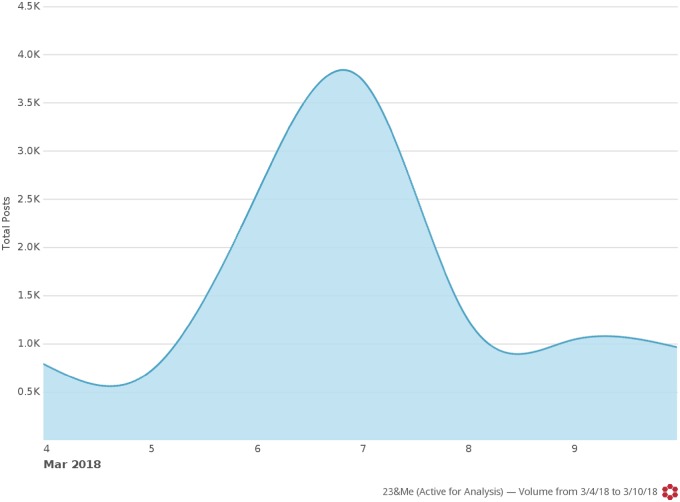
Twitter discourse about 23andMe during the week of the FDA’s authorization (Boston, MA, USA).

We identified 11 055 tweets about 23andMe across the week of FDA’s announcement. Twitter discourse about 23andMe and the FDA authorization peaked the day following their press release (7 March 2018; *n* = 3, 762) (Figure 1). The most influential Twitter accounts included large media outlets such as @CNN and @WSJ (Table 1).

**Table 1.** Top influencers among tweets identified during the study period

Author	Name	Country	Posts	Followers	Postdate GMT	Post text
@CNN	CNN	USA	172978	39629486	3/7	Genetic testing company 23andMe has been give federal approval to sell at-home kits that test for three breast cancer gene mutations https://t.co/6yBLXbVtekhttps://t.co/l8dwUEOLNj
@Reuters	Reuters Top News	USA	255347	19445229	3/6	U.S. FDA allows 23andMe to sell test for 3 mutations of breast cancer gene https://t.co/WqRm9LjYIGhttps://t.co/enEbD3e9Ti
@WSJ	The Wall Street Journal	USA	236287	15604458	3/9/	The FDA's approval of 23andMe’s cancer-risk test is the latest example of the agency’s course reversal. But will consumer access empower people—or confuse them? https://t.co/bCPKxK7wehhttps://t.co/XOaxdSNNsW
@cnni	CNN International	UK	148377	7610479	3/7	Genetic testing company 23andMe has been give federal approval to sell at-home kits that test for three breast cancer gene mutations https://t.co/vtyVgfsXx6https://t.co/dow9Oqy54q
@people	People	USA	201206	7883207	3/7	23andMe Kits Now FDA-Approved as a Genetic Test for Breast Cancer—But Is it Truly Accurate? https://t.co/Vrkrr38BhJ
@FortuneMagazine	FORTUNE	USA	161218	2294324	3/7	The new 23andMe FDA cancer screening approval is a game-changer for the company https://t.co/EicWJWx96j
@TechCrunch	TechCrunch	USA	175442	10140863	3/7	.@23andMe gets FDA green light for cancer risk test https://t.co/enbUle7k3b
@businessinsider	Business Insider	USA	473990	2364108	3/7	Genetic experts have a message for anyone thinking of taking 23andMe's new breast cancer test https://t.co/xEn5PqWBcUhttps://t.co/BVsQx3Utit
@newscientist	New Scientist	UK	49518	3374271	3/9	23andMe’s breast cancer test may create false sense of security https://t.co/URadJ5YD3hhttps://t.co/pw5Ob1XqPG
@sfchronicle	San Francisco Chronicle	USA	80776	145430	3/6	#23andMe can now sell breast cancer genetic test with no prescription needed https://t.co/gO2uTdzngL

The majority (67.8%, *n* = 5549) of discourse occurred in the United States, with the United Kingdom, and Canada comprising 6.1% and 3.4% of tweets, respectively (108 countries represented).

Most tweets (48.6%) were informational and 26.3% expressed opinions (about 23andMe and/or FDA authorization) or contained testimonials (personal experiences with genetic testing, 11.4%) (Table 2).

**Table 2.** Qualitative findings and exemplar tweets

	Frequency (%)				*P*-value	Example Tweet
	Overall	Before	During	After		
Type of Tweet					<.001	
Informational	294 (48.6)	19 (10.1)	145 (74.7)	130 (58.3)		RT @CNN Genetic testing company 23andMe has been give federal approval to sell at-home kits that test for three breast cancer gene mutations https://t.co/a45qCDZiwc 23andMe can now tell you about your breast cancer risk https://t.co/71qj5cc9sN
Opinion	90 (14.9)	43 (22.9)	13 (6.7)	34 (15.2)		RT @DanaFarber 23AndMe at-home cancer test: Should you take it? Dana-Farber's Huma Q. Rana, MD, weighs in: https://t.co/wHMFcLNJDi
Testimonial	69 (11.4)	35 (18.6)	15 (7.7)	19 (8.5)		Why I stood up for my health by taking the BRCA gene test #FoodAndDrugAdministration #BreastCancer #BrcaMutation #23andme #Mutation https://t.co/KjV32uyKaE I explored my DNA with 23andMe! Check them out @23andMe. https://t.co/VZ3TfVSbGS
Other (non-English, common language, NA)	152 (25.1)	91 (48.5)	21 (10.9)	40 (18.0)		Join our gene pool! 23andMe, Inc. is looking for Supply Chain Planner. Learn more or Jobvite a fr… https://t.co/AwKkXbaitE #job
Sentiment^a^					.46	
Positive	32 (20.1)	14 (17.9)	9 (32.1)	9 (17.0)		Thank you, @23andMe! “We will continue pioneering a path for greater access to health information, and promoting a more consumer-driven, preventative approach to health care.” Why I stood up for my health by taking the BRCA gene test #FoodAndDrugAdministration #BreastCancer #BrcaMutation #23andme #Mutation https://t.co/KjV32uyKaE
Neutral	58 (36.5)	28 (35.9)	8 (28.6)	22 (41.5)		I explored my DNA with 23andMe! Check them out @23andMe. https://t.co/TpkoQ6MEir 23&Me gets federal approval to inform people of breast cancer risk linked to three gene mutations https://t.co/H6hCjMCBm7 Related Lab Tests Online content https://t.co/9j4CVDO7Hz
Negative	69 (43.4)	36 (46.2)	11 (39.3)	22 (41.5)		23andMe’s breast cancer test may create false sense of security https://t.co/a622o85BGchttps://t.co/ylFUVAtrVv RT @GeneticCouns The #FDA approved a test today for #BRCA mutations, but it's important to know the test's limitations. Consumers should not make medical decisions based on the results without first consulting with a medical expert, such as a genetic counselor. Learn more: https://t.co/ougJ117hlJhttps://t.co/zpN7XLGghA

a Among opinion and testimonial tweets only; chi-square.

The types of tweets varied over the period (*P < *0.001); for example, 74.7% of tweets were informational on the day of the FDA announcement, versus 10.1% before and 58.3% following the announcement (Table 2). Among tweets expressing opinions or testimonials, 43.4% of tweets had a negative tone, 20.1% were positive, and the remaining were neutral. This sentiment (negative, positive, and neural) did not change across the time periods before, on the day of, or after the FDA announcement (*P* = .46). Tweets relaying opinions about 23andMe and the FDA announcement tended to be more negative than testimonials (78.3% vs 21.7%). Finally, we found 14.5% of tweets used 23andMe in everyday language, often as commentary about the ancestry, race, or ethnicity of an individual, group or oneself.

## DISCUSSION

Online discussion about 23andMe increased after the FDA’s authorization for the company to return *BRCA1/2* results for three specific pathogenic variants. Most of these tweets were informational in nature and were retweeted from news outlets. These tweets were neutral and informed the Twitter community about the FDA authorization (often referencing the FDA’s press release) and included discussion of the benefits and limitations of the authorized test. These findings align with other social media studies in which the majority of tweets about antimicrobial resistance were informational in nature,^9^ prominently from news sources.^10^ In contrast, a study about other health topics (elbow surgery, hereditary cancer) found that individual users from the general public^11^ and patient advocates and advocacy groups led discourse,^12^ demonstrating variation in how social media discourse rolls out by health topic. During our qualitative coding, we did not identify informational tweets that provided inaccurate information, suggesting that messaging by the FDA was largely adopted and shared via news outlets during the initial announcement of the authorization. Future studies should continue to monitor the accuracy of shared information over time and take a closer look at the accuracy of opinion pieces as well as user comprehension. In the case of the FDA authorization, tweets often provided additional information about potential harms of DTC genetic testing.

Tweets that shared opinions typically sought to inform the public regarding the potential harms of DTC testing. The most commonly referenced concern was that consumers may falsely assume that a negative 23andMe test result is definitive. Many of these tweets also discussed the need for consumers to consult with providers about their DTC genetic testing results, which aligns with recommendations by the American College of Medical Geneticists^13^ and public preferences.^14^ This suggests that with increasing demand for DTC testing, demand for genetic services follow-up may follow suit, and a continuing need to address the complex ethical, legal, and social implications of DTC testing. Testimonials were mostly positive in nature, often describing positive experiences with DTC testing or explaining why the new authorization may provide useful information for others. Interestingly, we did not find tweets during this period related to research; that is, no tweets provided data related to *BRCA1/2* testing, FDA authorizations or 23andMe. This finding may be due to the larger reach of news organizations relative to that of individual researchers. Moving forward, researchers and practitioners should consider partnering with news organizations to engage in social media discourse related to research findings about DTC testing. Through this partnership researchers and practitioners can engage the public in discussion about how DTC testing may impact health beliefs, behaviors, and outcomes.^8^ This will be increasingly important in this era of precision medicine and DTC genetic testing.

While this study provides the first snapshot of social media discourse around the FDA authorization of *BRCA1/2* testing through 23andMe, it is not without limitations. First, while we sought to use comprehensive and broad search terminology, relevant tweets may have been excluded or misclassified during qualitative coding. Future research should examine not only how information about the FDA authorization was disseminated on Twitter, but also how information sharing impacts knowledge, beliefs, and behaviors.

In conclusion, we see that information about the FDA’s authorization of DTC for three pathogenic *BRCA1/2* variants greatly increased discussion of DTC genetic testing. As more genetic technologies are brought to the DTC market, social media sites, such as Twitter, and news outlets will play an important role in disseminating this information, providing a platform for information exchange, consumer testimonials, opinion pieces, and research.
